# Development of an intervention tool for precision oral self-care: Personalized and evidence-based practice for patients with periodontal disease

**DOI:** 10.1371/journal.pone.0225453

**Published:** 2019-11-21

**Authors:** Wen-Jen Chang, Shih-Yin Lo, Chen-Li Kuo, Yen-Li Wang, Hsu-Chuan Hsiao

**Affiliations:** 1 Department of Information Management, Chang Gung University, Taoyuan, Taiwan; 2 Department of Dentistry, Chang Gung Memorial Hospital, Taoyuan, Taiwan; 3 Department of Periodontics, Chang Gung Memorial Hospital, Taoyuan, Taiwan; 4 Department of Nursing, Chang Gung Memorial Hospital, Taoyuan, Taiwan; Centre Hospitalier Regional Universitaire de Tours, FRANCE

## Abstract

**Background:**

Oral self-care plays an important role in maintaining oral health and preventing the occurrence of oral diseases. The association between good oral care and good oral hygiene is well known. However, the adherence to a proper daily oral hygiene regimen generally remains poor, so the prevalence of oral diseases remains high. Periodontal disease is the most common oral disease in the population. To enhance the adherence to good oral hygiene behaviors for patients with periodontal disease, we developed a personalized and evidence-based mobile application as an intervention tool for the purpose of initiating and improving good oral self-care.

**Objective:**

The objective of this study was to demonstrate the systematic development process and content of the oral self-care mobile application, OSCA.

**Methods:**

The systematic development process of OSCA consists of three phases: behavioral diagnosis, intervention design, and assessment of OSCA. Firstly, behavioral problem of oral self-care was identified by the experts in periodontics care. Secondly, the intervention functions and the mode of delivery were designed based on the capability-opportunity-motivation behavioral model, which is the underpinning model behind the behavior change wheel framework. Thirdly, the developed app was evaluated by the experts through a heuristics evaluation checklist by adopting Morville’s Honeycomb model, and the final version of OSCA was assessed by the patients with periodontal disease using the System Usability Scale (SUS).

**Results:**

The problems of target behavior were identified and incorporated into the design of intervention functions. For the beta version of the OSCA, experts proposed four main suggestions to improve the usefulness. Experts evaluated the beta and final versions of the app using a heuristics evaluation checklist, providing mean scores of 4.38 and 4.62, respectively. For usability testing, 87 participants completed both the specified tasks and the SUS questionnaire, providing an SUS median score of 77.5 (IQR = 12.5) and an overall mean completion time of 12.22 minutes for the specified tasks. The mean scores of the intervention functions for capability establishment, motivation enhancement, and opportunity creation were 6.13, 5.88, and 6.06, respectively.

**Conclusions:**

The study presents a rigorous design process of developing an evidence-based and personalized mobile application for oral self-care. The results of the expert evaluation confirmed the validated design and the participants were satisfied with the designed app.

## Introduction

Oral health is essential for the maintenance of general health and well-being at every stage of life. Good oral hygiene results in a mouth that looks and smells healthy and not only enables nutrition of the physical body, but also enhances social interaction and promotes self-esteem and feelings of well-being [1]. Oral health is frequently affected on a daily basis by various oral diseases, such as dental caries, periodontal disease, and even oral cancer [2].

Oral diseases with high prevalence are one of the most common public health issues worldwide. A high proportion of the world’s population (approximately 90%) suffers from oral diseases at some point in their lives [3]. Periodontal disease and dental caries are the most common chronic diseases and cause severe pain in later stages [2,4]. Almost 100% of adults and 60%–90% of schoolchildren worldwide suffer from dental caries, and about 20%–50% of the global population suffers from periodontal disease [5]. Severe periodontitis is the sixth most prevalent disease in the world, which may lead to tooth loss [5,6]. The overall prevalence of severe periodontitis is 11.2%, which increases with age, and the incidence steeply rises in adults aged 30–40 years [6]. Furthermore, there is a strong correlation between oral diseases and the main non-communicable diseases such as diabetes and cardiovascular diseases [3,7]. Therefore, oral diseases are considered the most important global oral health burdens [2].

The economic impact of oral diseases is quite huge. WHO estimated that oral diseases are the fourth-most expensive diseases to treat in most industrialized countries [7]. The impact of oral diseases on the global economy consists of direct and indirect treatment costs. The estimated totaling worldwide costs because of oral diseases were at US$442 billion in 2010 [8] and increased to US$544.41 billion in 2015 [9]. In addition, oral diseases have a large impact on people’s daily lives and are responsible for the yearly loss of millions of school and work hours around the world [8,10–11]. Productivity losses due to work absence from dental diseases were estimated at US$144 billion worldwide in 2010 [8]. From an economic point of view, the improvement in the oral health of the population may be highly beneficial and could further increase people’s well-being [9].

Effective self-performed regular oral hygiene has been identified as a key attribute in the oral diseases prevention [12–13]. During the clinical encounters, the dental care team will provide advice and instruction on oral health to the patients based on the results of their clinical examinations to develop their oral self-care skills for maintaining good oral hygiene [14]. However, patients’ adherence to a proper daily oral hygiene regimen generally remains poor [15–17]. A large number of adults clean their teeth less than the recommended time [18] and adults have been shown to have problems achieving oral cleanliness through self-performed oral hygiene. Increasing the adherence to oral hygiene behavior is regarded as an essential attribute in the prevention of dental caries and periodontal disease [19–20].

Lack of self-regulatory skills is associated with a disinclination to change health behaviors, including deficits in self-efficacy, planning, and action control. Behavior change interventions have been employed in previous studies to translate behavior intention into action for the maintenance of health-enhancing behaviors [18,21–22]. The adoption of behavior change interventions can enhance an individual’s ability to perform oral self-care as well as long-term dental habits critical for the maintenance of oral hygiene, which are key factors for achieving good oral health [18,23].

Smartphone use has increased rapidly in recent years worldwide. There were over 2 billion smartphone users globally in 2016 and this value is estimated to have reached one-third of the world’s population by 2018 [24]. In addition to this rapid growth in smartphone use, the smartphone characteristics of mobility and popularity provide an unprecedented opportunity for applications targeting health and health-related behaviors. The features of mobile devices (such as smartphones) make them particularly appropriate for providing individual-level support for health care, facilitates temporal synchronization of intervention delivery, and enables interventions to claim people’s attention at the most relevant time [25]. For a large percentage of smartphone users, mobile health applications (mHealth apps) are promising tools for engaging patients in their own health care. mHealth apps can be used as a powerful behavior change tool for health prevention and self-management as they are ubiquitous and can be carried on the person. The enormous impact of mHealth apps on many important health-related domains such as chronic disease management, mental health, and patient education has been reported [25–26].

As mHealth apps continue to proliferate, the effectiveness of the apps is becoming increasingly important. However, there are still significant barriers to expanding the role and efficacy of mHealth apps to improve the health of consumers and ultimately the population [23,25–26]. To ensure the effectiveness of mHealth apps, incorporating the needs of their intended users into the design process is important [27–28]. With adequate consideration of user needs, the developed apps will be perceived as more useful and easy to use.

Well-designed behavior change interventions are fundamental to enhance the effective practice of clinical medicine and public health. The behavior change wheel (BCW) framework, which can be employed to achieve a more efficient design for interventions, was developed to support users in behavior change [29]. Delivering personalized and helpful information can increase patients’ engagement in self-care, but not yet incorporated into the existing oral care app.

To improve oral hygiene behaviors for patients with periodontal disease, we developed a personalized oral self-care app (OSCA) based on evidence-based practice by adopting the BCW framework. This study aims to provide a detailed description of the development and content of OSCA by adopting the BCW framework.

## Methods

The personalized OSCA was created with evidence-based data and knowledge of clinical practice. This study consisted of three phases: (1) diagnosis of behavior problems, (2) analysis and design of intervention functions, (3) assessment of the developed application, consisted of expert evaluation for both the beta and final versions of the app, and patients with periodontal disease participated in the usability testing for the final version of OSCA.

### Phase I: Behavioral diagnosis

Behavioral problems for patients with periodontal disease in performing oral self-care were identified by two periodontists and one head nurse of Dental Services Department. Behavioral diagnosis was used to capture the problem of maintaining oral hygiene for the patients, and behavior targeting focused on extracting the appropriate action to achieve good oral self-care, including knowledge and skills. In this phase, we identified the features of the target behavior, which is the desired behavior to enhance the patients’ oral hygiene.

During dental visits, patients receive advice and are educated on how to adequately perform oral self-care by the dental care team in accordance with the findings of clinic examinations. The instructions are aimed at improving the patients’ knowledge and skill in good daily oral cleaning practices to improve their oral hygiene. However, a high proportion of patients still cannot maintain adequate oral hygiene. The behavioral issue is the dental care team provides too much information for the patients and the message may not all be understood by patients. To fully clarify the barriers and facilitating factors of the target behaviors is important for the next step of designing the intervention functions.

In the “behavior system,” capability, opportunity, and motivation (COM) interact to generate behavior that in turn influences these components. In other words, the COM-B model requires three essential conditions (capability, opportunity, and motivation) for the target behavior to occur [29]. We developed intervention functions using this model.

### Phase II: Intervention function design

Having identified the target behavior, intervention functions were created to link the behavior system necessary for the target behavior to occur. The intervention functions of the OSCA were designed to encourage patients to achieve good ability for oral self-care based on the identified target behaviors. The BCW framework provides guidance on which types of intervention functions are more likely to initiate the target behavior for each component of the COM-B model [29]. With consideration to the essential conditions for the COM-B model, there are three components for designing interventional functions aimed at generating the desired behavior: establishing capability, providing opportunity, and enhancing motivation.

#### Establishing capability

*Capability* is defined as an individual’s psychological and physical ability to engage in the activity concerned, including the necessary knowledge and skills [29]. To improve patients’ oral self-care capabilities, dentists provide instructions during dental visits aimed at improving patients’ oral hygiene knowledge and skills. However, after receiving such important information, the most common barrier to good oral self-care is that patients usually do not recall all of the advice and guidance they received from the dental care team [21,30]. To reduce the burden on the patients’ memory, the most important information necessary for patients to perform proper daily oral hygiene was identified by periodontal care professionals and the result was employed for the design of practical functions of intervention. The intervention functions were designed in accordance with the principles of being understandable, specifiable, and accessible to alleviate the burden of memory for patients. The intervention functions of establishing capability for daily oral care were divided into two parts: generalized and individualized information. The former was provided through on-line videos and the latter was presented graphically to help patients with recall. Intervention functions of establishing capability were designed to provide knowledge and oral self-care skills and patients could view the information anytime and anywhere.

#### Enhancing motivation

*Motivation* is defined as brain processes that energize and direct behavior, including habitual processes, emotional responses, and analytical decision-making [29]. Motivational intervention is broadly defined as any clinical strategy designed to enhance patient motivation for change [19,23,31].

Capability establishment for patients with periodontal disease is the most important aspect of this study, and the functions of motivational intervention were created to enhance patient intention to engage in the target behavior. In designing the intervention functions for enhancing motivation, two specific cognitions were adopted to enhance the likelihood of patients to achieve the target behavior. These were emphasizing the importance of performing the target behavior and establishing the patient’s confidence with using the designed app.

#### Creating opportunity

*Opportunity* is defined as all the factors that lie outside the individual’s control that make the behavior possible or an individual feel capable of undertaking the new behavior [29]. Motivation is simply the intention or desire to engage in the target behavior and is not sufficient to create behavior change; hence, there is a gap between motivation and behavior [21]. Furnishing intentions with planning actions diminishes the gap between intentions and behavior. Creating opportunities for patients to transform motivation into action makes it possible to achieve behavioral change. Studies have indicated that making plans to engage in a behavior has a significant impact on behavior change [19,32–33]. In addition, the function of self-monitoring serves as one of the most important behavioral strategies for raising awareness of the initiation of a behavior change journey and has long been recognized as the key to success in many areas of behavior change, including diabetes and blood pressure control, obesity management, and physical activity monitoring [23,27].

Implementation intention and action planning interventions have been shown to be effective in changing diverse behaviors such as physical activity promotion, dietary management, cancer screening, stopping smoking, and dental health behaviors [33]. In this study, the intervention functions of planning and self-monitoring were developed to create opportunities for patients to engage in good oral self-care actions. The function of planning intervention was used to encourage patients to create a specific plan to execute the target behaviors. The function of self-monitoring was developed to increase the awareness of performing good oral self-care and to initiate the behavior change journey.

### Phase III: Assessment of the developed application

The OSCA was developed to reflect the designed intervention functions and the usability was evaluated to identify the problems with the app and create a useful and easy to use interface. A usability assessment was conducted for tasks orientation and was divided into two stages: expert evaluation with suggested modification for the beta version of the app and usability testing of the final version.

#### Expert evaluation

To examine the usability problems of the designed app, we employed Morville’s Honeycomb model of user experience [34] to create a *heuristics evaluation checklist* for expert evaluation. Honeycomb model is a well-established and extremely popular visualization tool, which depicts 7 aspects of the user experience. The created *heuristics evaluation checklist* consisted of 11 questions as shown in Table 1.

**Table 1:** Checklist of heuristic evaluation for experts assessing OSCA.

Facets	Checklist for the usability problem
Usable	1. Easy to use. 2. Loading speed of the dental chart is fast.
Useful	3. Content will be used frequently. 4. Content is categorized appropriately.
Desirable	5. Information are integrated as visual images. 6. Specific information are provided personalized.
Findable	7. The information of how to use the App is provided. 8. The menu can be found easily.
Accessible	9. The provided information of oral care can be easily accessed.
Credible	10. Zero errors occurred while using it. 11. Information captured from this App can be trusted.

Five experts participated in the stage of the usability assessment, since Nielsen reported that the best results for usability assessment come from testing 3–5 evaluators [35]. Three of the experts are informaticians with experience in interface design and/or human–computer interaction, and the other two are periodontists with more than 10 years of clinical experience. A description of the full functionality of the app was given to the experts and each expert was asked to independently complete the given tasks (Table 2). According to the operating experience of the app, the experts were asked to complete the *heuristics evaluation checklist* (Table 1) for the usability problems. Each expert independently evaluated the app using a 5-point Likert-scale ranging from *strongly disagree* (1) to *strongly agree* (5). Regarding the experts’ experiences while using the app, their opinions on what they liked and which aspects could be improved were also collected.

**Table 2:** Tasks for evaluating the usability of the created app.

1. Please read the app user manual.
2. Please set a tooth brushing remainder at 9pm
3. Please open the dental chart and find the areas required extra attention.
4. Please run the brushing mode once.
5. Please find the oral care video menu.
6. Please watch a video on “how to use dental floss” or “how to use an interdental brush”.
7. Please find the brushing reminder that you set earlier.

**Fig 1:**
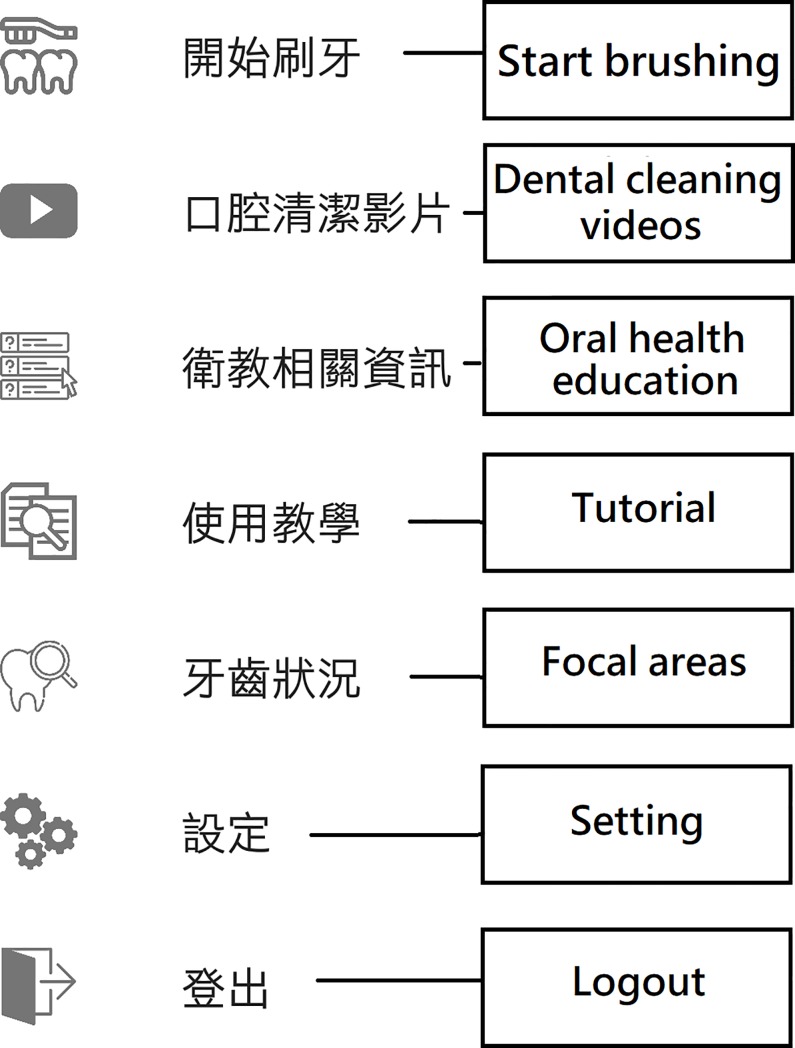
The main menu of OSCA.

**Fig 2:**
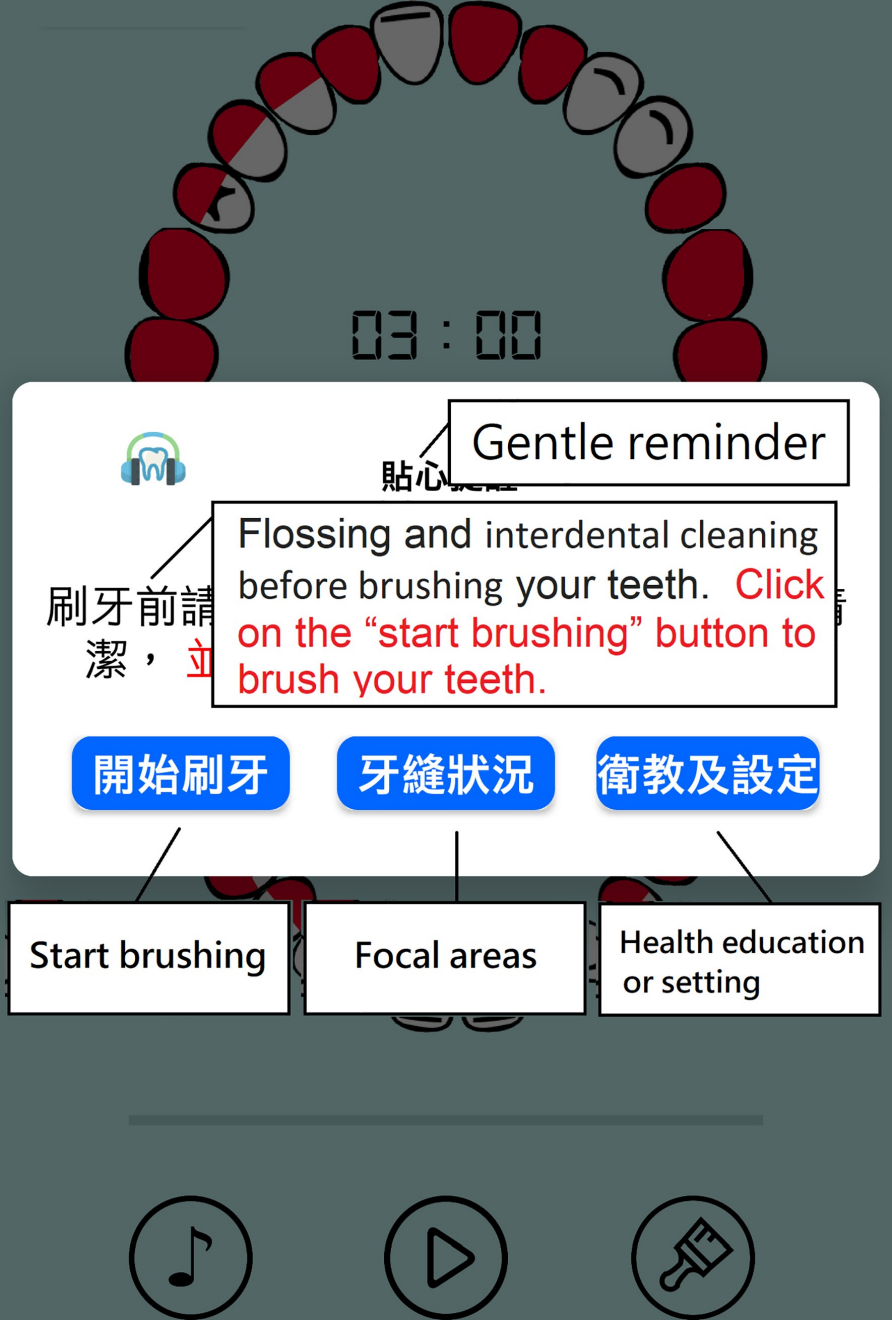
A popup window reminds the users to use dental floss or an interdental brush to clean interdental areas before brushing their teeth.

**Fig 3:**
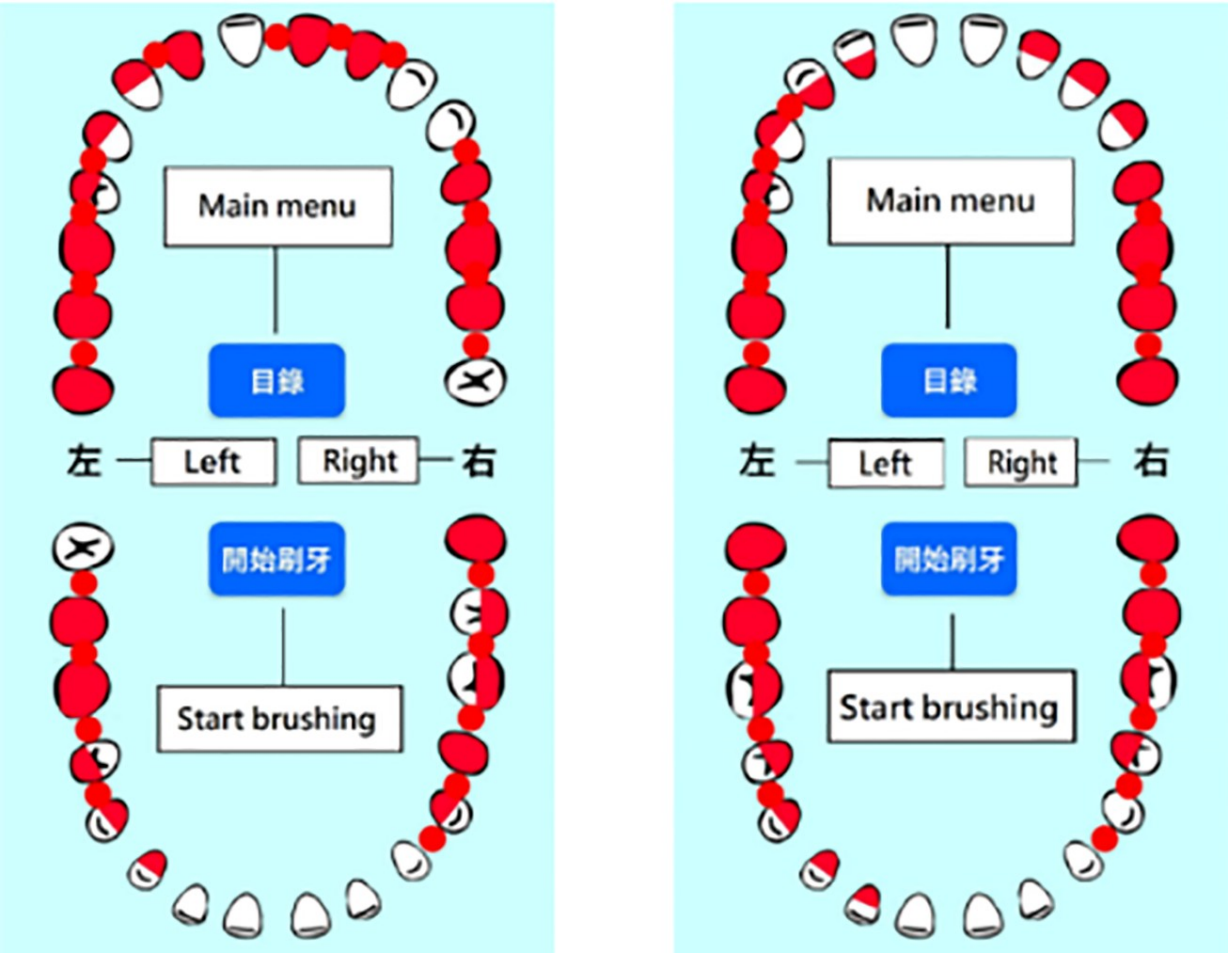
The focal areas that require extra cleaning effort.

**Fig 4:**
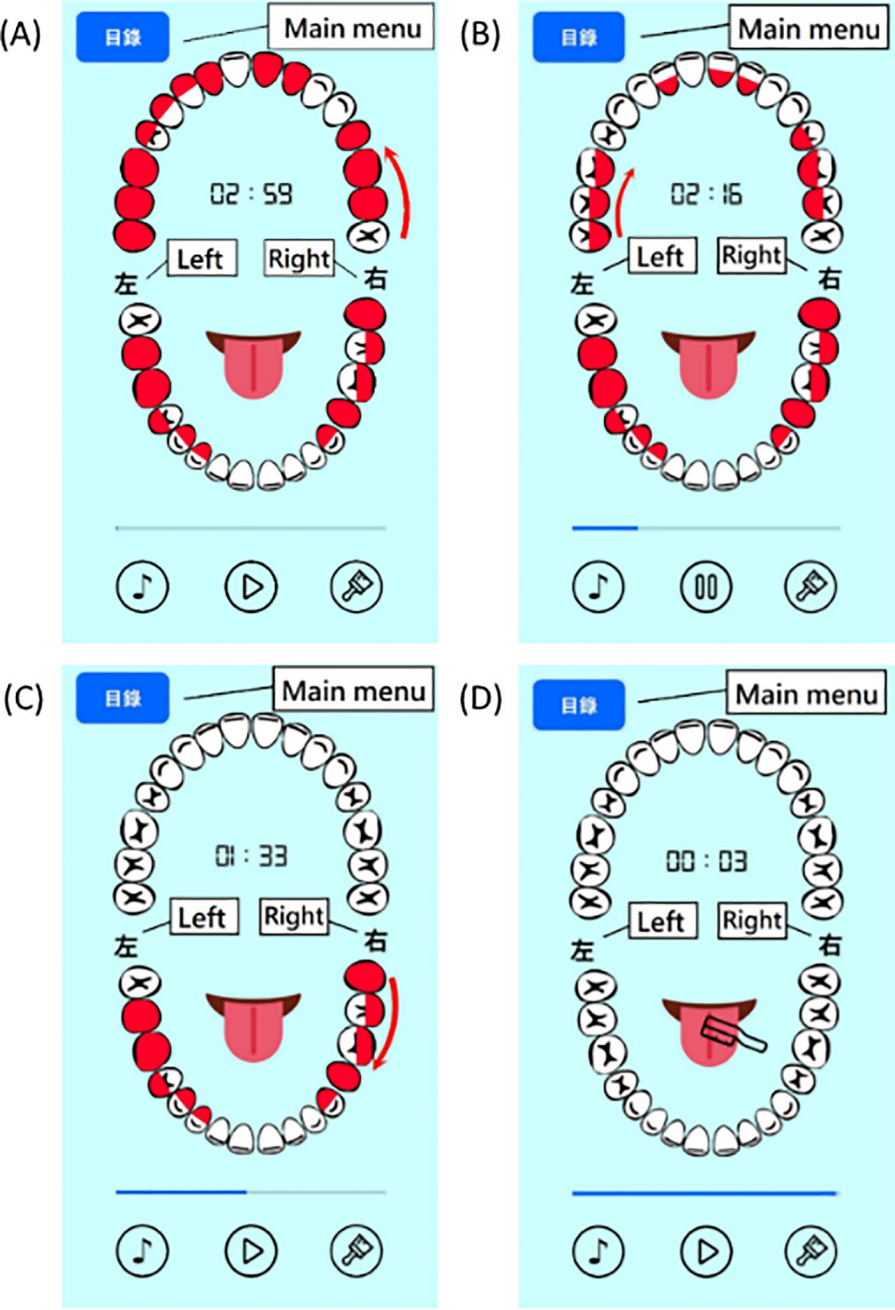
A sequence of screenshots showing the progress of tooth brushing.

The suggestions for modifying the beta version app were based on the collective opinions of the five experts, and the app was accordingly modified. A demonstration of the final version of the app was given to the experts and a consensus was obtained before usability testing. The screen shots of OSCA are shown in Figs 1–4. The paired sample t-test was used to compare the experts’ evaluation of the beta and final versions of the app.

#### Usability testing

For the usability test, the participants were recruited from April to October 2018 from periodontics clinics. The inclusion criteria required that the participants were smartphone users, at least 20 years of age, and able to perform oral self-care. The participants were told that participation was voluntary, and all information disclosed would be confidential. Before participating in the investigation, the subjects provided written informed consent. For completing the usability evaluation with a self-administered questionnaire, the participants were compensated with a coupon worth US $3.5. Ethical approval was obtained from the Institutional Review Board of Chang Gung Memorial Hospital, Taoyuan, Taiwan (201701853B0).

The periodontist highlighted the focal areas that require extra cleaning effort in the dental chart according to the result of the patient’s clinical examination. A research assistant introduced the features of OSCA and demonstrated the specified tasks in person. The tasks demonstrated by the research assistant were the same as those employed during the expert evaluation phase (Table 2). After demonstration by the research assistant, the participants practiced the tasks repeatedly and were asked to complete the specified tasks independently after confirming they could comfortably perform the tasks. If the participant was able to successfully complete the tasks, the completion time was recorded and they were then asked to complete a questionnaire.

Following testing participants were asked to complete a questionnaire consisting of four parts. The first part was the demographic variables of the respondents (including gender, age, and education level), the second part was the habitual variables (including daily oral hygiene habits and perceived dental health), the third part was the usability testing, and questions for the favorability assessment of OSCA were placed at the end.

For usability testing, the System Usability Scale (SUS) [36] which has been proven to be robust and versatile [37–38] was adopted. The SUS was developed by Brooke [36] to address a strong need in the usability community for a tool that could quickly and easily collect a user’s subjective rating of a product’s usability. The SUS questionnaire consists of 10 items in the form of statements (Table 3) that have to be rated on a 5-point Likert-scale ranging from *strongly disagree* (1) to *strongly agree* (5). Scoring the SUS is somewhat complicated because of the alternating tone of the items (positive- and negative-tone). The first step is to derive the score contributions from the raw item scores. For the odd-numbered items which are positive-tone items, subtract 1 from the raw score, and for the even-numbered items which are negative-tone items, subtract the raw score from 5. Compute the sum of the score contributions of the 10 items, and then multiply by 2.5 to get the standard SUS score. For each individual, the SUS score ranges from 0 to 100 [38].

**Table 3:** Measures used in the usability testing.

1. I think that I would like to use this app frequently.
2. I found the app unnecessarily complex.
3. I thought the app was easy to use.
4. I think that I would need the support of a technical person to be able to use this app.
5. I found the various functions in this app were well integrated.
6. I thought there was too much inconsistency in this app.
7. I would imagine that most people would learn to use this app very quickly.
8. I found the app very cumbersome to use.
9. I felt very confident using the app.
10. I needed to learn a lot of things before I could get going with this app.

The favorability assessment of OSCA comprises individual evaluation of intervention functions and overall evaluation of the app. The assessment of individual functional favorability required the participants to evaluate the intervention functions of the app, which can be categorized into capability establishment, motivation enhancement, and opportunity creation based on the essential conditions of the COM-B model [29]. The overall favorability, defined as likeability, was used to assess the extent to which the participants liked the app. The items for the likeability assessment were adapted from Hoj et al [39]. There were 13 and 5 assessment items for the individual functions and likeability of the app, respectively. A 7-point Likert response scale was used to measure these items, ranging from *strongly disagree* (1) to *strongly agree* (7).

## Results

From the diagnosis of the target behavior, lack of knowledge and incorrect skills of daily oral care were found to be barriers to maintaining good oral health for the patients with periodontal disease. Advice and instructions on oral self-care for improving oral health was provided to the patients based on the clinical examinations. However, we found that it’s difficult for patients to recall all the advice and agree actions about oral self-care provided by the dental care team.

Several practical tips to improve patients’ memory for oral self-care were incorporated into the design of intervention functions for OSCA based on the behavior diagnosis. The designed intervention functions were categorized based on the COM-B model. To establish capability, the advice provided by the dental care team and information on oral self-care were integrated into the designed app: (1) Providing oral care videos, (2) Pointing out the focal areas that require extra cleaning effort in the dental chart, (3) Showing the progress of tooth brushing with an arrow to direct the user to brush his/her teeth in the same order. To create opportunity, four intervention functions were applied: (1) A reminder to use dental floss and an interdental brush to clean teeth, (2) Customized settings for reminders for daily oral care, (3) A reminder to clean teeth at least twice a day, (4) A reminder to brush teeth according to the time set. To enhance motivation, five intervention functions were employed: (1) Providing a user manual, (2) Rewarding the participant with applause upon completion of a tooth brushing session, (3) Providing important information about periodontal disease, (4) Making the indicator disappear when the user finished brushing the focal areas, (5) Playing music during the tooth brushing session.

### Expert evaluation

The mean scores of the experts’ evaluation from the *heuristics evaluation checklist* was 4.38 (shown in Table 4) and only one was item rated less than 4, namely “loading speed for dental chart is fast (3.6).” To improve the loading speed, we redesigned the dental chart to reduce the file size. Additionally, to enhance the acceptance and usefulness of the app, the experts recommended some modifications for the beta version of the app.

**Table 4:** Evaluation results for experts assessing OSCA.

Facets	Checklist for the usability problem	Beta version	Final version
Usable	1. Easy to use.	4.6	4.8
	2. Loading speed of the dental chart is fast.	3.6	4.2
Useful	3. Content will be used frequently.	4.6	4.6
	4. Content is categorized appropriately.	4.2	4.6
Desirable	5. Information are integrated as visual images.	4.8	4.8
	6. Specific information are provided personalized.	4.8	5.0
Findable	7. The information of how to use the App is provided.	5.0	5.0
	8. The menu can be found easily.	4.8	4.8
Accessible	9. The provided information of oral care can be easily accessed.	4.2	4.2
Credible	10. Zero errors occurred while using it.	4.0	4.4
	11. Information captured from this App can be trusted.	4.2	4.6
Valuable	12. Providing important information for oral self-care.	4.0	4.6
	13. An appropriate medium for recalling the advice of oral self-care which provided by the dental care team.	4.2	4.4
	Aggregate	4.38	4.62

First, two experts suggested that the focal areas that require more thorough cleaning be presented in red rather than in black. The focal area, which consists of teeth stained a red color after a plaque test, usually accumulates large masses of bacteria to form dental plaque. According to the results of the clinical plaque test, the dentist will advise patients that red-stained areas should receive extra care to improve their oral hygiene. The focal area was highlighted in black in the beta version of the app, but one expert suggested emphasizing the focal area with a red color to illustrate the test result more realistically.

Second, two experts proposed that the movement of the guiding arrow showing the brushing progress should be further segmented. The mouth was separated into quadrants in the beta version of the app, with one arrow for each quadrant directing the user on how to brush his/her teeth. Dentists recommend brushing a group of 2–3 teeth at a time and going around the mouth in a circle to get every tooth. Therefore, the experts suggested that the guiding arrow should also move around a width of 2–3 teeth at a time, such that the user can follow the dentist's recommendations to brush his/her teeth appropriately.

Third, one expert recommended that the red indicators should disappear when the guiding arrow moved past the focal area. This recommendation is based on the linking of implementation intentions and the behavior change technique [31]. Implementation intentions connect an intention to behave in a specified manner within a particular context, thus, creating a situation-specific cue to perform the behavior would increase the engagement and enhance the probability of behavior change. [40]. In the beta version of the app, the focal area was constantly highlighted in black (changed to red in the final version). To enhance the vivid presentation of the brushing progress, the expert recommended removing the indicator as the guiding arrow moved past the focal area to provide a situation-specific cue to the user to follow the app to brush his/her teeth.

Finally, the experts suggested enlarging the dental chart used to show the progress of tooth brushing to improve the readability. The dental chart in the beta version took up only 50% of the screen. The size of the model scale up to utilize the configuration of the mobile phone screen effectively.

Experts use the *heuristics evaluation checklist* again to assess the final version of OSCA. The result was shown in Table 4 and the mean scores for the final version app of OSCA was 4.62.

### Usability testing

One hundred and three patients who were smartphone users willing to use an app to assist their oral self-care were recruited to participate in the usability test. Of the 103 recruited patients, 4 participants who could not complete the tasks due to unfamiliarity with the use of the mobile application, and 12 participants who did not fully complete the questionnaire were excluded. Eighty seven of these participants completed both the specified tasks and the SUS questionnaire. Table 5 shows the demographic characteristics of these 87 participants. Most of the participants (30/87, 34.5%) were between the ages of 45 and 54 years, with men comprising 58.6% (51/87) of the subjects. The mean completion time for the specified tasks was 10.36 minutes (95% CI, 9.7–11.03 minutes) for respondents under the age of 35 years, and increased with increasing age. The mean time to complete the specific tasks for the respondents aged no less than 65 years was 14.89 minutes (95% CI, 14.11–15.67 minutes).

**Table 5:** Demographics and the SUS score (n = 87).

Demographics		n (%)	SUS score (Mean ± SD)
**Gender**			
	Male	51 (58.6)	78.09 ± 10.51
	Female	36 (41.4)	77.43 ± 9.48
**Age, in years**			
	< 35	8 (9.2)	75.94 ± 12.95
	35–44	20 (23.0)	80.00 ± 10.82
	45–54	30 (34.5)	78.50 ± 8.92
	55–64	19 (21.8)	78.42 ± 9.55
	65 or older	10 (11.5)	71.75 ± 9.51
**Education level**			
	Less than high school	8 (9.2)	75.63 ± 9.80
	High school	25 (28.7)	76.90 ± 9.77
	College or university up to bachelor	48 (55.2)	78.75 ± 10.35
	College or university up to master or PhD	6 (6.9)	77.08 ± 10.66

The mean SUS score (n = 87) was 77.8. The quartiles for the SUS score were 72.5, 77.5, and 85, respectively. The descriptive statistics of the SUS scores for demographic variables are presented in column three in Table 5. The mean SUS score was highest in the age group for 35–44-year olds (80.0) and then decreased with increasing age. Elderly participants aged 65 and above had a mean SUS score of 71.75. There was no difference in the mean SUS scores between the age groups after one-way ANOVA (p-value = 0.243). The mean scores for the three categories of the designed intervention functions, capability establishment, motivation enhancement, and opportunity creation, were 6.13, 5.88, and 6.06, respectively.

Regarding the favorability of intervention functions (shown in Table 6), most respondents reported that they liked the intervention functions of the app. More than half of the respondents strongly agreed and agreed that they liked the intervention functions, with the exception of “rewarded with applause when three minutes of brushing was achieved (49.4%).” The favorability proportion of “strongly agree” and “agree” for the “built-in function of playing music while brushing” was 58.6%. For the other 11 evaluations of the designed intervention functions, the proportions of “strongly agree” and “agree” were above 70%. Three items were rated as “strongly agree” and “agree” by more than 80% of the respondents, specifically “demonstrate the relative interdental position that needs to be cleaned thoroughly,” “guided arrow for the movement of the toothbrush,” and “as the guiding arrow for the toothbrush movement passes by, the red mark indicating the area that requires extensive cleaning disappears.”

**Table 6:** Responses regarding the favorability of intervention functions.

Item			n (%)						
	Category of COM-B	Mean	Strongly disagree	Disagree	Slightly disagree	Neutral	Slightly agree	Agree	Strongly agree
IF1. I like the oral care video, which make me learn more about oral cleaning.	Capability	5.97	1 (1.1)			13 (14.9)	6 (6.9)	33 (37.9)	34 (39.1)
IF2. I like the App pointing out the focal areas in my mouth that require extra care.	Capability	6.10				10 (11.5)	8 (9.2)	32 (36.8)	37 (42.5)
IF3. I like the App showing the focal areas that require extra cleaning effort in the dental chart.	Capability	6.18				8 (9.2)	8 (9.2)	31 (35.6)	40 (46.0)
IF4. I like the App reminding me to using dental floss and an interdental brush to clean my teeth.	Opportunity	6.07		2 (2.3)		7 (8.0)	10 (11.5)	30 (34.5)	38 (43.7)
IF5. I like the App user manual, it makes the App easy-to-use.	Motivation	6.09				7 (8.0)	12 (13.8)	34 (39.1)	34 (39.1)
IF6. I like the App rewarding with applause when achieving a tooth brushing session.	Motivation	5.41			1 (1.1)	26 (29.9)	17 (19.5)	22 (25.3)	21 (24.1)
IF7. I like the app providing important information about periodontitis disease.	Motivation	5.97				9 (10.3)	15 (17.2)	33 (37.9)	30 (34.5)
IF8. I like the App allowing me to set the reminders for tooth brushing.	Opportunity	6.00				10 (11.5)	12 (13.8)	33 (37.9)	32 (36.8)
IF9. I like the App reminding me to brush my teeth according to the time that is set.	Opportunity	5.97			1 (1.1)	10 (11.5)	13 (14.9)	30 (34.5)	33 (37.9)
IF10.I like the App reminding me to clean my teeth at least twice a day.	Opportunity	6.18				6 (6.9)	12 (13.8)	29 (33.3)	40 (46.0)
IF11.I like the App showing the progress of tooth brushing with an arrow, which directs me to brush my teeth in the same order.	Capability	6.25				5 (5.7)	8 (9.2)	34 (39.1)	40 (46.0)
IF12.I like the App making the red indicators disappear when I have finished brushing the focal areas.	Motivation	6.25				5 (5.7)	9 (10.3)	32 (36.8)	41 (47.1)
IF13.I like the background music played in the App during the tooth brushing session.	Motivation	5.69				17 (19.5)	19 (21.8)	25 (28.7)	26 (29.9)

### Overall favorability

Regarding the app likeability (Table 7), most respondents reported that the app was useful (34.5%, 30/87, “strongly agree” and 35.6%, 31/87, “agree”). Similarly, 35.6% strongly agreed and 32.2% agreed that the app was easy to use. More than 90% of respondents reported they would enjoy using the app for daily oral self-care (36.8%, 32/87, “strongly agree”; 31.0%, 27/87, “agree”; and 23.0%, 20/87 “slightly agree”). A majority of respondents indicated they liked the app (29.9% “strongly agree,” 34.5% “agree,” and 23.0% “slightly agree”), and respondents had similar “strongly agree,” “agree,” and “slightly agree” response rates for their willingness to recommend the app to others.

**Table 7:** Responses regarding the overall likeability of OSCA.

Item	Mean	Strongly disagree	Disagree	Slightly disagree	Neutral	Slightly agree	Agree	Strongly agree
1. The app was useful.	5.93				10 (11.5)	16 (18.4)	31 (35.6)	30 (34.5)
2. This app was easy to use.	5.90			1 (1.1)	10 (11.5)	17 (19.5)	28 (32.2)	31 (35.6)
3. I would enjoyed using the App for my daily oral self-care.	5.93		1 (1.1)		7 (8.0)	20 (23.0)	27 (31.0)	32 (36.8)
4. I liked the app.	5.79		1 (1.1)		10 (11.5)	20 (23.0)	30 (34.5)	26 (29.9)
5. I would recommend the app to others.	5.76		1 (1.1)		12 (13.8)	20 (23.0)	27 (31.0)	27 (31.0)

## Discussion

In this study, we proposed a rigorous design to develop a personalized oral self-care app based on information obtained from clinical practice. The study was divided into three phases. The first phase was behavioral diagnosis, which was necessary to identify inappropriate oral self-care behaviors. Second, intervention functions were designed to inspire users to achieve the target behavior. Interventions developed based on theory are more likely to be effective [41]; therefore, we adopted the COM-B model, which is the underpinning of BCW framework [29] to design the intervention functions. Finally, the usability of the developed app was assessed in two stages: expert evaluation for both the beta and final versions of the app and usability testing for the final version of the app by the patients with periodontal disease.

In the expert evaluation stage, experts evaluated the beta version of the app with a *heuristics evaluation checklist* and only one of thirteen items was rated less than 4 out of 5. The experts proposed four modifications to facilitate user engagement with the app. The beta version of the app was modified accordingly and the final version was developed. A paired sample t-test was used to comparing the experts’ evaluation of the beta and the final versions of the app, the mean usability score of the final version (4.62) was significant better than that of the beta version (4.38) with a p-value of 0.0086. This result indicated that the final version was perceived to be significantly better than the beta version.

For the usability test, the mean SUS score reported by the participants without assistance was 77.8, which is higher than the minimum value of 70 used to indicate good usability [37]. The intervention functions of OSCA can be categorized into capability establishment, motivation enhancement, and opportunity creation based on the essential conditions of the COM-B model. The participants provided high favorability ratings for OSCA (the mean favorability scores for the three components of establishing capability, enhancing motivation, and creating opportunity were 6.13, 5.88, and 6.05, respectively).

Good oral care capabilities can help individuals maintain good oral health. Patients with periodontal disease usually have insufficient knowledge or incorrect skills regarding oral self-care. Clinical examination and appropriate therapy for periodontal disease can improve patients’ oral health and alleviating their discomfort. However, periodontal therapy can only be successful when a high level of self-performed oral hygiene is combined with professional maintenance care [42]. In this study, OSCA was designed for patients with periodontal disease to establish good capabilities to perform oral self-care, including general knowledge, oral care skills, and personalized advice from a professional dental care team based on clinical examinations. The participants rated the designed intervention functions for establishing capability as 6.13 out of 7. The result showed that the participants had a high favorability for the intervention functions of capability establishment provided by the OSCA, especially for providing important personal information on the basis of a clinical examination to reduce the user's memory load. In the literature, Misra et al indicated that patients do not always recall as much advice and agree actions about future oral care which were recommended by their dentists [30]. Additionally, Newton & Asimakopoulou pointed out that dental terms or phrases taken for granted in dentistry may not be understood by patients [21]. Therefore, providing important information in an appropriate form to establish capability for oral self-care is necessary. To make the information more understandable, intervention functions were designed to deliver important information about daily oral self-care through a dental chart and links to on-line videos. Another clinical finding related to dental self-care is patients are often habitually concentrated in a certain area when brushing their teeth, causing plaque or periodontitis to occur in some areas. To overcome this problem, the progress of tooth brushing was depicted with an arrow to direct users to brush their teeth methodically so that the brushing time can be evenly distributed in each area of the teeth.

Motivation is regarded as an important driver of behavior change [43]. Arweiler et al [42] reported that the capability and motivation of patients to maintain good oral hygiene are the foundations for the long-term success of periodontal treatment. The motivational intervention functions for oral self-care were designed to provide important information about periodontal disease, provide a visualization of the OSCA user manual and motivate patients to perform proper oral care. By investigating the motivational intervention functions, we found that the functions of “playing music during the tooth brushing session” and “rewarding with applause when a tooth brushing session was achieved” received relatively lower ratings. “Rewarded with applause when three minutes of brushing was achieved” received the lowest score, with a proportion of “agree” and “strongly agree” below 50%. This may indicate that the incentive of this function is relatively low in terms of patient perception compared to the other intervention functions. Regarding the functional evaluation of “playing music during the tooth brushing session,” the proportions of participant ratings for “agree” and “strongly agree” were relatively low (58.6%) compared to ratings for the other intervention functions, with the exception of “rewarded with applause when three minutes of brushing was achieved.” However, some participants reported that brushing teeth with music would be funny and some indicated that it would motivated them to actually brush their teeth for 3 minutes. The built-in function of playing music while brushing has been referenced in the literature. Clemens and Taylor proposed the idea of using music to motivate brushing for longer periods [44] and Underwood et al indicated that the development of mobile devices with music functions has become more practical [45].

Motivation alone is not sufficient to create behavior change and creating opportunity can increase the likelihood of transforming the motivation into action [30]. Planning an approach substantially increases the likelihood of behavior change. Interventions for planning have been adopted in healthcare studies [30,32] and are regarded as an effective approach to enable people to translate their “good” intentions into action. Furthermore, interventions incorporating elements of goal setting, planning the behavior, and monitoring the behavior (GPS elements) were reported to be effective in creating behavior change [46]. To put motivation into action, we integrated GPS elements into the intervention design to create opportunities for patients to implement oral self-care, including setting goals to brush the teeth at least twice a day, planning for possible times to perform oral cleaning through setting a reminder, and self-monitoring to brush the teeth in a sequential order lasting up to 3 minutes. The mean favorability of the creating opportunity feature was 6.055 out of 7, indicating that the participants were satisfied with the development of planning interventions.

### Overall favorability

The participants gave high ratings for the overall likeability of OSCA. This may be because OSCA was developed as an evidence-based and personalized oral self-care app. It provides personalized and important information to assist patients with daily oral self-care. A high proportion of the participants reported that they would recommend the app to others, and more than half of the participants (62/103) requested and installed OSCA on their smartphones after the usability test. Five of the respondents requested the installation of OSCA on the smartphones of their family members. This result may indicate that OSCA can be used as a good intervention tool to encourage periodontal patients to perform daily oral self-care.

### Limitations

Several limitations to our study should be considered. This study focused on describing the designing process and the content of an evidence-based and personalized oral self-care app. We studied the OSCA in a usability testing environment, not in a real-world environment. Additionally, the effectiveness of OSCA for improving patients’ oral health was not investigated. A future study should explore the behavior change outcome for evaluating the effectiveness of OSCA to assist patients in performing oral self-care by measuring the plaque index.

## Conclusion

With the diagnosis of target behavior, we found that lack of knowledge, incorrect skills, and providing too much information about oral care to patients are the barriers to improving their oral health. In this study, we incorporated several practical tips into the design of intervention functions for OSCA based on the COM-B model. The designed intervention functions were categorized into establishing capability, creating opportunity, and enhancing motivation. The developed app of oral self-care was assessed and resulted in good usability through expert evaluation for both the beta and final versions of the app and usability testing of the final version by the patients with periodontal disease. This study presented a rigorous design process for developing an evidence-based and personalized mobile application for oral self-care, making it a unique app for improving oral health in patients with periodontal disease.

## Supporting information

S1 Data Data for expert evaluation. (XLSX)

S2 Data Data for usability testing. (XLSX)

S1 File Supporting information_survey questions. (DOCX)
