# Supplementary material for: Development of an intervention tool for precision oral self-care: Personalized and evidence-based practice for patients with periodontal disease
Source: PLoS One. 2019 Nov 21;14(11):e0225453. doi: 10.1371/journal.pone.0225453 (PMC6874065; doi:10.1371/journal.pone.0225453)
Supplement: S1 File — (DOCX) [file pone.0225453.s003.docx]

**Survey Questions Translated into English**

**Part A. Demographic variables**

1. Gender: □ Male; □Female
2. Age, in years: □ < 35; □ 35-44; □ 45-54; □ 55-64; □ 65 or older
3. Education level: □ Less than high school; □ High school; □College or university up to bachelor; □College or university up to master or PhD

**Part B. Habitual variables：**

1. I brush my teeth when I wake up and before going to bed every day.
2. I clean teeth (brush or rinse with water) after meals.
3. I use floss to clean my teeth every day.
4. I use interdental brush to clean my teeth every day.
5. I think regular oral cleaning (brushing and interdental cleaning) is important.
6. I think I have good oral health.

**Part C. The System Usability Scale**

1. I think that I would like to use this app frequently.

2. I found the app unnecessarily complex.

3. I thought the app was easy to use.

4. I think that I would need the support of a technical person to be able to use this app.

5. I found the various functions in this app were well integrated.

6. I thought there was too much inconsistency in this app.

7. I would imagine that most people would learn to use this app very quickly.

8. I found the app very cumbersome to use.

9. I felt very confident using the app.

10. I needed to learn a lot of things before I could get going with this app.

**Part D.**

- 1. I like the oral care video, which make me learn more about oral cleaning.
  2. I like the App pointing out the focal areas in my mouth that require extra care.
  3. I like the App showing the focal areas that require extra cleaning effort in the dental chart.
  4. I like the App reminding me to using dental floss and an interdental brush to clean my teeth.
  5. I like the App user manual, it makes the App easy-to-use.
  6. I like the App rewarding with applause when achieving a tooth brushing session.
  7. I like the app providing important information about periodontitis disease.
  8. I like the App allowing me to set the reminders for tooth brushing.
  9. I like the App reminding me to brush my teeth according to the time that is set.
  10. I like the App reminding me to clean my teeth at least twice a day.
  11. 11.I like the App showing the progress of tooth brushing with an arrow, which directs me to brush my teeth in the same order.
  12. I like the App making the red indicators disappear when I have finished brushing the focal areas.
  13. I like the background music played in the App during the tooth brushing session.
  14. The app was useful.
  15. This app was easy to use.
  16. I would enjoy using the App for my daily oral self-care.
  17. I liked the app.
  18. I would recommend the app to others.

**Survey Questions in Original Language (Chinese)**

**Part A. 基本資料：**

1. 性別：□男；□女
2. 年齡：□ <35歲；□ 35-44歲；□ 45-54歲；□ 55-64歲；□ 65歲以上
3. 教育程度：□國中(含)以下；□高中(職)；□大學(專)；□研究所(含)以上

**Part B. 口腔清潔習慣：**

1. 我每天起床及睡前都有刷牙的習慣
2. 我在用餐後，有清潔牙齒(刷牙或漱口)的習慣
3. 我每天有使用牙線清潔牙縫
4. 我每天有使用牙間刷清潔牙縫
5. 我認為有規律的口腔清潔(刷牙及清潔牙縫)是重要的
6. 我認為我的牙齒狀況很好

**Part C. APP易用性評估：**

1. 我想我會願意經常使用這款APP。
2. 我覺得這款APP過於複雜。
3. 我認為這款APP很容易使用。
4. 我想我需要有人幫助才能使用這款APP。
5. 我覺得這款APP的功能整合得很好。
6. 我覺得這款APP有太多不一致的地方。
7. 我可以想像大部份的人很快就可以學會使用這款APP。
8. 我覺得這款APP使用起來很麻煩。
9. 我很有自信能使用這款APP。
10. 我需要學會很多額外的資訊，才能使用這款APP。

**Part D. 口腔保健APP之喜好評估**

1. 我喜歡此APP提供的口腔清潔衛教影片，讓我更清楚知道如何進行口腔清潔。
2. 我喜歡此APP能指出我個人在進行口腔清潔時需加強的地方。
3. 我喜歡此APP透過牙齒模型圖片，呈現我要加強清潔的位置。
4. 我喜歡此APP能提醒我要使用牙線及牙間刷進行口腔清潔。
5. 我喜歡此APP的使用教學，讓我可以很容易上手使用。
6. 我喜歡此APP在我完成刷牙時播放的喝采聲。
7. 我喜歡此APP會讓我在刷牙後，能有牙齒清新的美好感覺。
8. 我喜歡此APP能讓我自行設定可能的刷牙時間。
9. 我喜歡此APP在預設的刷牙時間，提醒我要記得刷牙。
10. 我喜歡此APP能提醒每日至少要進行2次的口腔清潔。
11. 我喜歡此APP在刷牙時，以箭頭指示刷牙進度，讓我可依序刷牙。
12. 我喜歡此APP在我刷牙經過須加強注意的地方時，紅色標示也會隨著消失。
13. 我喜歡此APP能提供背景音樂，讓我在刷牙時能專心刷牙。
14. 我覺得這款APP很有用。
15. 我喜歡這款APP。
16. 為提升口腔清潔，我會願意繼續使用這款APP。
17. 我會向其他人推薦這款APP。
18. 整體而言，我非常想使用這款APP。
